# Career Plans Among Graduating US Emergency Medicine Residents

**DOI:** 10.1001/jamanetworkopen.2025.55376

**Published:** 2026-01-27

**Authors:** Dave W. Lu, Bo Gu, D. Mark Courtney, Bryan G. Kane, Michelle D. Lall, Michael Gottlieb, Yvette Calderon, Christopher Bennett

**Affiliations:** 1 Department of Emergency Medicine, University of Washington School of Medicine, Seattle; 2 Quantitative Sciences Unit, Department of Medicine, Stanford University, Palo Alto, California; 3 Department of Emergency Medicine, UT Southwestern Medical Center, Dallas, Texas; 4 Department of Emergency Medicine, Lehigh Valley Health Network, Allentown, Pennsylvania; 5 Department of Emergency Medicine, Emory University School of Medicine, Atlanta, Georgia; 6 Department of Emergency Medicine, Rush University Medical Center, Chicago, Illinois; 7 American Board of Emergency Medicine, East Lansing, Michigan; 8 Department of Emergency Medicine, Thomas Jefferson University, Philadelphia, Pennsylvania; 9 Department of Emergency Medicine, Stanford University, Palo Alto, California

## Abstract

**Importance:**

The emergency medicine (EM) workforce is undergoing complex change, including a potential projected physician surplus, the volatile and evolving interests of medical students, and the continued high rates of burnout. Understanding the career intentions of graduating residents is critical to inform workforce planning.

**Objective:**

To examine the career plans, anticipated career durations, and key factors associated with the career decisions of graduating US EM residents.

**Design, Setting, and Participants:**

This cross-sectional survey study, which sampled graduating residents from all Accreditation Council for Graduate Medical Education–accredited categorical EM programs, was conducted after the 2023 American Board of Emergency Medicine In-Training Examination. Resident-level characteristics, including gender, race and ethnicity, educational debt, and burnout status, were examined. Data were obtained from June 20 to August 18, 2024, and analysis occurred from December 16, 2024, to August 12, 2025.

**Main Outcomes and Measures:**

Primary outcomes included immediate postresidency career plans, intended practice setting, anticipated years of clinical practice, and importance of 10 career decision factors.

**Results:**

Of 2782 eligible residents, 2711 (97.4%; 1394 men [51.4%]) responded to at least 1 survey item. Most respondents (1654 [61.0%]) reported educational debt exceeding $100 000; 1964 of 2239 respondents (72.4%) met criteria for burnout. Immediate career plans included community-based practice (852 [31.4%]), fellowship (631 [23.3%]), hybrid (community-based hospital affiliated with an academic medical center) (416 [15.3%]), and academic EM (161 [5.9%]). Most residents (1597 [58.9%]) intended to work in an urban setting, with 652 (24.1%) planning to work in a rural setting. The mean (SD) anticipated duration of clinical EM practice was 22.4 (8.3) years. The most important career decision factors were lifestyle (2135 of 2189 [97.5%]), geographic location (1930 of 2176 [88.7%]), professional fulfillment (1906 of 2173 [87.7%]), and salary expectations (1808 of 2183 [82.8%]). Female residents were more likely than male residents to report professional fulfillment and burnout as important factors and less likely to prioritize salary expectations. Residents with $200 000 or more in debt (1376 [50.7%]) were significantly more likely to cite salary as a major factor ($200 000-$299 000: odds ratio [OR], 2.01 [95% CI, 1.39-2.89]; $300 000-$399 000: OR, 2.31 [95% CI, 1.58-3.39]; ≥$400 000: OR, 1.58 [95% CI, 1.05-2.42]).

**Conclusions and Relevance:**

In this survey study of graduating EM residents in 2023, most respondents planned to practice clinical EM for 2 decades primarily in community or hybrid settings, with a minority of residents interested in rural practice. Career decisions are overarching and encompass career factors; lifestyle, location, professional fulfillment, and salary were the strongest career factors. Burnout, educational debt, and demographic characteristics were associated with career decisions. These findings have implications for workforce modeling, retention strategies, and policies to support the EM workforce.

## Key Points

**Question:**

What factors are associated with the anticipated career plans and durations of practice among graduating US emergency medicine (EM) residents?

**Findings:**

In this national survey study of 2711 EM residents, 46.7% planned to pursue a community-based or hybrid practice, with 24.1% intending to work in rural settings. The most important factors in career decisions were lifestyle (97.5%), geographic location (88.7%), professional fulfillment (87.7%), and salary (82.8%).

**Meaning:**

Understanding the career intentions and motivations of a recent cohort of graduating residents can inform workforce planning, policy development, and targeted interventions to support sustainable EM career trajectories.

## Teaser

This survey study examines the career plans, anticipated practice durations, and key factors associated with the career decisions of graduating US emergency medicine residents.

## Introduction

Over the past 5 years, the specialty of emergency medicine (EM) has experienced noteworthy changes that have affected the emergency physician (EP) workforce. A widely publicized 2021 study projecting a potential surplus of more than 7800 EPs by 2030 has been extensively debated in the medical literature and EM community.^1,2^ Augmenting this debate is the subsequent volatile and evolving interest of US medical students in pursuing EM, leading to the 2022 and 2023 EM Match results with an unexpectedly large number (7.5% and 18.4%, respectively) of unmatched positions.^3,4^ These developments took place in the aftermath of the COVID-19 pandemic, when trainees witnessed significant clinical education disruptions,^5,6^ an increase in the burnout and attrition of clinicians,^7^ and record-level hospital crowding and boarding.^8^

Although prior work has explored some of the factors associated with EM residents’ career plans,^9,10,11,12^ little is known about how recent graduating EM residents are planning their careers during this tumultuous time. Their decisions about practice type, setting, and anticipated duration of clinical practice may inform future EM workforce considerations and projections. Understanding these dynamics is important because EM remains a critical safety net and front door to care for patients and communities across the health care system. As the volume of patients admitted to emergency departments (EDs) increases and as more patients with primary care needs, chronic care needs, and unscheduled acute care needs present to EDs, the role of EPs has expanded to include inpatient observation, hospital-at-home care, remote patient monitoring, and ED-based critical care.^13^

The primary objective of this study was to examine the career plans of a national cohort of graduating EM residents. A secondary study objective was to investigate which factors were most important to them when making career decisions.

## Methods

### Study Design, Setting, and Selection of Participants

This was a cross-sectional survey study of the career plans of a national cohort of graduating EM residents. An electronic survey was administered after the 2023 American Board of Emergency Medicine (ABEM) In-Training Examination (ITE), an annual computer-based examination taken by residents training in Accreditation Council for Graduate Medical Education (ACGME)–accredited EM residency programs. Resident participation was voluntary, all data were deidentified, and additional written or oral consent was waived by ABEM. Only residents graduating in 2023 who were enrolled in categorical, ACGME-accredited EM programs in the US were included in the analysis. Data were obtained from June 20 to August 18, 2024. The University of Washington institutional review board deemed this study to be exempt given that all data were deidentified. We followed the Strengthening the Reporting of Observational Studies in Epidemiology (STROBE) and American Association for Public Opinion Research (AAPOR) reporting guidelines.^14,15^

### Outcome Measures and Variables

Demographic information collected through survey questions included self-reported gender, race and ethnicity (American Indian or Alaska Native; Asian; Black or African American; Hispanic, Latino, or Spanish origin; Middle Eastern or North African; Native Hawaiian or Other Pacific Islander; White; multiracial; unknown; or other [any category or subcategories not already indicated]), and educational debt (eAppendix in Supplement 1). Self-identified race and ethnicity data were collected to describe the study population, assess representativeness, and allow evaluation of potential differences across groups. Information on clinical postgraduate year and 3- or 4-year program format was provided by the ABEM and matched with each survey response.

### Burnout

Burnout was measured using the abbreviated Copenhagen Burnout Inventory, a 6-item tool that assesses the internal and external factors of burnout.^16^ Residents who reported internal, external, or both factors of burnout were considered to have burnout.

### Career Plans

Residents were asked their immediate career plans after residency training. Options included fellowship, community practice, academics, hybrid of community-based practice affiliated with an academic medical center, or other (eg, Veterans Affairs hospital, urgent care setting, active military practice, and *locum tenens*). Practice setting was queried dichotomously using the US Department of Agriculture definition of urban as a metropolitan area core with a population greater than 50 000 people, with the definition of rural as 50 000 people or less.^17^

Residents were also asked to estimate the anticipated duration of their clinical careers by entering a numerical value to the following question: “How many years do you expect to practice clinical EM?” In addition, residents were asked, “When you cut back or leave clinical EM, what do you plan to do?” Response options included nontraditional EM clinical practice (eg, urgent care, telemedicine, and observational medicine), administration, education, research, obtaining additional medical education or training, working in a different career other than medicine, retiring, and other.

### Factors Considered Important in Career Choice

Items from the Association of American Medical Colleges Graduation Questionnaire^18^ were adapted to ask residents how important each of the following 10 factors were in their career choice: lifestyle, competitiveness or prestige of career path, mentor or role model influence, salary expectations, family expectations, geographic location, burnout, professional fulfillment, prospect of a surplus of EM physicians by 2030, and the COVID-19 pandemic. Residents indicated the degree of influence of each factor via a Likert scale (strong, moderate, minor, or none), with responses dichotomized into major (responses of strong and moderate influence) and minor influence (responses of minor or no influence).

### Statistical Analysis

All statistical analyses and data visualization were conducted from December 16, 2024, to August 12, 2025, using R, version 4.4.1 (R Project for Statistical Computing). Prior to analysis, data were validated to ensure accurate coding of survey responses; this involved confirming that numerical values corresponded correctly to their respective survey options (eg, 1 = a, 2 = b). Data were then cleaned and renamed, excluding anomalies in continuous variables (eg, such as nonsensical responses of −30 or 999 years) and collapsing a subset of categorical variables (eg, collapsing gender and race and ethnicity into fewer categories) to address small cell sizes to improve statistical stability and ensure sufficient power for group comparisons. Analyses comparing outcomes by gender were restricted to respondents identifying as male or female; for race and ethnicity, categories of White and all other race (American Indian or Alaska Native; Asian; Black or African American; Hispanic, Latino, or Spanish origin; Middle Eastern or North African; multiracial; Native Hawaiian or Other Pacific Islander; unknown; and other) were created. We aimed for complete case analysis by excluding missing values unless the proportion of missingness was extremely high (>25%) or missing patterns of key variables were not random.

Descriptive statistics were computed to summarize demographic characteristics, career plans, and burnout measures. As appropriate, continuous variables were reported as mean (SD) values, median (IQR) values, and ranges. Categorical variables were presented as frequencies and proportions. In secondary analyses, linear regression modeling was used to examine the associations among demographic factors, educational debt, burnout, and expected years in clinical practice. Multivariate modeling included variables such as gender, race and ethnicity, program format, and educational debt, with output reported as odds ratios (ORs) and 95% CIs. All statistical tests were 2-tailed, with a significance threshold of *P* < .05.

## Results

Of the 2782 graduating EM residents in the 2022-2023 academic year,^19^ 2711 (97.4%; 1394 men [51.4%], 912 women [33.6%], and 24 nonbinary individuals [0.9%]; American Indian or Alaska Native, 9 [0.3%]; Asian, 347 [12.8%]; Black or African American, 114 [4.2%]; Hispanic, Latino, or Spanish origin, 158 [5.8%]; Middle Eastern or North African, 59 [2.2%]; Native Hawaiian or Other Pacific Islander, 5 [0.2%]; White, 1464 [54.0%]; multiracial, 75 [2.8%]; unknown, 14 [0.5%]; and other race or ethnicity, 52 [1.9%]) completed at least 1 portion of the survey (Table 1). Most respondents (1654 [61.0%]) reported educational debt exceeding $100 000. Of the 2239 residents who completed the burnout portion of the survey, 1964 (72.4%) met criteria for burnout.

**Table 1.** Respondent Characteristics

Characteristic	Respondents, No. (%) (N = 2711)
Gender	
Male	1394 (51.4)
Female	912 (33.6)
Nonbinary	24 (0.9)
Missing	381 (14.1)
Race and ethnicity	
American Indian or Alaska Native	9 (0.3)
Asian	347 (12.8)
Black or African American	114 (4.2)
Hispanic, Latino, or Spanish origin	158 (5.8)
Middle Eastern or North African	59 (2.2)
Multiracial	75 (2.8)
Native Hawaiian or Other Pacific Islander	5 (0.2)
Unknown	14 (0.5)
White	1464 (54.0)
Other^a^	52 (1.9)
Missing	414 (15.3)
Program format	
PGY 1-3	2088 (77.0)
PGY 1-4	623 (23.0)
Educational debt, $	
None	348 (12.8)
<100 000	199 (7.3)
100 000-199 999	278 (10.3)
200 000-299 999	556 (20.5)
300 000-399 999	526 (19.4)
≥400 000	294 (10.8)
Missing	510 (18.8)

Abbreviation: PGY, postgraduate year.

^a^ Includes any category or subcategories not already indicated (survey in the eAppendix in Supplement 1).

**Figure 1.**
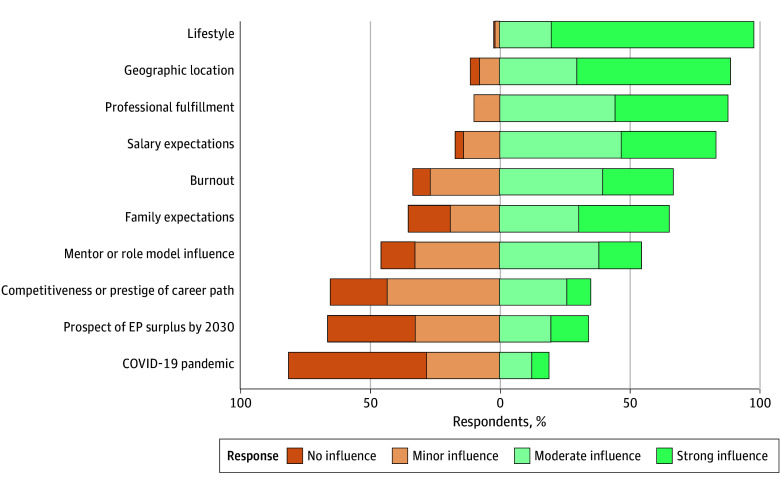
Factors Considered Important in Career Choices Missing responses ranged from 522 of 2711 (19.3%) to 538 of 2711 (19.8%). EP indicates emergency physician.

In terms of immediate career plans after graduation, most respondents planned to practice primarily in a community-based hospital (852 [31.4%]) or in a hybrid environment (416 [15.3%]) (Table 2). Overall, 1597 respondents (58.9%) planned to practice in an urban setting vs 652 (24.1%) in a rural setting. Seven of the 10 factors assessed for potential association with career decisions were considered by most respondents to have a major influence. Lifestyle was cited most frequently (97.5% [2135 of 2189]), followed by geographic location (88.7% [1930 of 2176]), professional fulfillment (87.7% [1906 of 2173]), and salary expectations (82.8% [1808 of 2183]) (Figure 1). In contrast, the COVID-19 pandemic (18.9% [412 of 2179]) followed by the prospect of a potential surplus of EM physicians by 2030 (33.9% [740 of 2180]) had the least influence on residents’ career decisions.

**Table 2.** Resident Career Plans and Setting

Plan or setting	Respondents, No. (%) (N = 2711)
Career plan	
Fellowship or additional training	631 (23.3)
Academic medical center	161 (5.9)
Community-based hospital affiliated with an academic medical center	416 (15.3)
Community-based hospital	852 (31.4)
Other^a^	158 (5.8)
Unsure	41 (1.5)
Missing	452 (16.7)
Career setting	
Urban	1597 (58.9)
Rural	652 (24.1)
Missing	462 (17.0)

^a^ Veterans Affairs hospital, urgent care setting, active military practice, *locum tenens*.

When examining the associations between resident characteristics and each of the 7 factors identified to be important, several significant findings were uncovered (eFigures 1-7 in Supplement 1). Residents from 4-year programs were less likely to choose a rural setting (OR, 0.70 [95% CI, 0.55-0.88]) compared with those from 3-year programs, whereas White residents were more likely to choose a rural setting than their peers from other racial and ethnic groups (OR, 1.76 [95% CI, 1.43-2.16]) (eFigure 2 in Supplement 1). Residents with $200 000 or more of educational debt (1376 [50.7%]) were more likely to report the importance of salary expectations in their career choices than residents with less debt ($200 000-$299 000: OR, 2.01 [95% CI, 1.39-2.89]; $300 000-$399 000: OR, 2.31 [95% CI, 1.58-3.39]; ≥$400 000: OR, 1.58 [95% CI, 1.05-2.42]) (eFigure 4 in Supplement 1). Finally, female residents were less likely than male residents to report the importance of salary (OR, 0.69 [95% CI, 0.54-0.87]) (eFigure 4 in Supplement 1) and more likely to report the importance of professional fulfillment (OR, 1.73 [95% CI, 1.30-2.32]) (eFigure 3 in Supplement 1) and burnout (OR, 1.40 [95% CI, 1.16-1.70]) (eFigure 5 in Supplement 1) in their career decisions.

**Figure 2.**
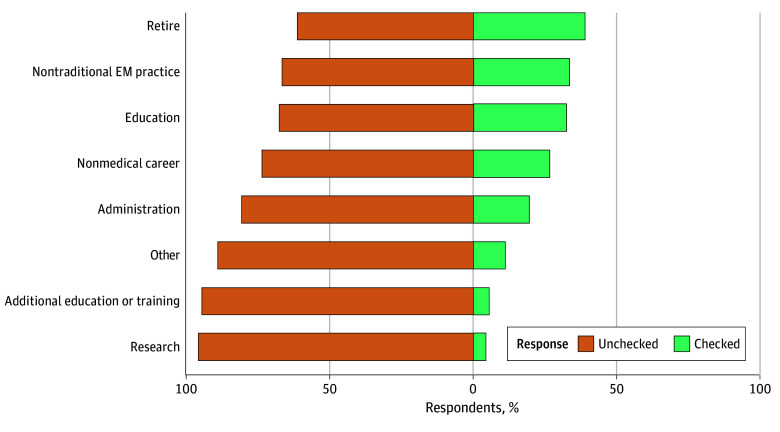
Resident Plans After Cutting Back or Leaving Clinical Emergency Medicine (EM) Missing responses, 539 of 2711 (19.9%).

Graduating residents reported that they expected to practice clinical EM for a mean (SD) of 22.4 (8.3) years. When residents choose to cut back or leave clinical EM, their most common anticipated plans were to retire (1048 of 2711 [38.7%]), work in a nontraditional EM practice (904 of 2711 [33.3%]), or engage in education (876 of 2711 [32.3%]) (Figure 2). When investigating the associations between resident characteristics and the anticipated number of years in clinical practice, there were no significant differences in the expected duration of clinical practice by gender, program format, or educational debt (Table 3). White residents expected to practice clinical EM 2.1 (95% CI, 1.4-2.9) years longer than residents from other racial and ethnic groups, and residents with burnout expected to practice clinical EM 5.1 (95% CI, 4.0-6.2) years longer than residents without burnout.

**Table 3.** Resident Characteristics and Anticipated Number of Years in Clinical Practice^a^

Characteristic	β (95% CI)	*P* value
Gender		
Male	NA	>.99
Female	−0.02 (−0.72 to 0.69)	
Race and ethnicity		
All other race	NA	<.001
White	2.1 (1.4 to 2.9)	
Program format		
PGY 1-3	NA	.80
PGY 1-4	−0.13 (−0.95 to 0.70)	
Educational debt, $		
None	NA	
<100 000	0.07 (−1.4 to 1.5)	>.99
100 000-199 000	−0.35 (−1.6 to 0.96)	.60
200 000-299 000	−0.18 (−1.3 to 0.93)	.80
300 000-399 000	−0.71 (−1.8 to 0.41)	.20
>400 000	0.17 (−1.1 to 1.5)	.80
Burnout		
No	NA	<.001
Yes	5.1 (4.0 to 6.2)	

Abbreviation: PGY, postgraduate year.

^a^ Missing responses, 608 of 2711 (22.4%).

## Discussion

This survey study of graduating EM residents identified several key findings regarding the factors that were associated with their career choices. Nearly half of graduating EM residents planned to practice primarily in a community-based setting or in a hybrid environment, and most also planned to work in an urban rather than rural setting. Among the factors that were associated with career decisions, most residents reported that lifestyle, geography, professional fulfillment, and salary were the most important. Recent developments, such as the COVID-19 pandemic and the prospect of a potential surplus of EM physicians, were not considered important by most residents. Lastly, graduating residents expected to practice clinical EM for approximately 2 decades.

A 2020 study demonstrated that most practicing EPs were located in urban (92%) rather than rural (8%) areas.^17^ In addition, residents who trained in urban areas were more likely to practice in urban environments after completion of residency.^20^ It has been suggested that these findings may be because of residents’ personal preference to live in urban areas, but it has also been hypothesized that residents’ relative discomfort working in resource-limited areas due to lack of exposure during their training may dissuade them from choosing to practice in a rural setting.^20^ In our cohort of residents graduating in 2023, a larger proportion of them (24.1%) than in the 2020 study reported plans of practicing in a rural setting. We do not know if this level of reported interest is ultimately consistent with what residents chose on graduation and, if so, how long they will remain practicing in a rural setting. We also do not know if this relatively high proportion of residents who plan to practice in a rural setting suggests potential increasing interest in rural EM or if it reflects trends in job market financial incentives.^21^ Most recent estimates showed that less than half of the rural EM workforce consisted of EPs, with advanced practice providers replacing the shortfall.^1^ Physicians in rural EDs are also older than their urban peers, with the number of EPs leaving the rural workforce outpacing those entering.^1^ It will be important for future work to track residents’ plans to practice rural EM given the significant need for EM-trained and board-certified EPs in rural EM to address current shortages and inequities in access.

Previous work among EM residents revealed that male and female residents prioritized different factors in their job search. Compared with male residents, female residents in a 2019 study were more likely to consider factors such as parental leave policy, desired practice type and setting, desired location, and patient population to be served as very important.^22^ In contrast, those researchers found that male residents were significantly more likely than female residents to consider salary as very important (51.8% vs 29.6%). Our results appeared to be consistent with these findings, with female residents more likely than male residents to report the importance of professional fulfillment and burnout and less likely to report the importance of salary expectations in their career decisions. It remains unclear if the lower emphasis placed on salary expectations by female EM residents is associated with well-documented gender disparities in compensation in EM.^23^ However, it raises the possibility that gender-based differences in job selection priorities may be associated with persistent salary disparities, particularly if female residents are more likely to choose lower-paying positions that better align with their personal or professional values.

Our study showed that most EM residents graduated with high levels of educational debt. We found that residents with $200 000 or more of educational debt were more likely than residents with less debt to report the importance of salary expectations in their career choices. This finding is consistent with prior research that showed residents with higher levels of debt were less likely to choose a fellowship or an academic career on graduation, particularly for men, given that fellowship training and working for a nonprofit institution were associated with lower incomes.^23,24^ Future work should continue to address the issue of educational debt (eg, via the creation or expansion of loan repayment incentives) so that the career choices that residents make may be more aligned with what truly fulfills them long term, rather than what addresses more immediate financial pressures.

Residents in our study reported that they planned to practice clinical EM for approximately 2 decades after completion of their training. Given that the median age of residents in 2023 was 30 years,^25^ this projection is consistent with recent work showing that the median age of EPs who exhibited attrition in 2019 was 49.7 years.^26^ This projection also appears to confirm that EPs retire from clinical practice approximately 1 decade earlier than previously suggested in studies of physicians across multiple specialties.^27^ Future studies should further explore potential reasons (eg, physical demands, burnout, and circadian rhythm challenges of shift work) for EPs’ relatively early retirement age. Our study found no differences in anticipated years of practice by gender, whereas the aforementioned study found that the median age of attrition for male EPs (53.5 years) was significantly greater than that for female EPs (43.7 years).^26^ This discrepancy suggests that after female EPs begin independent practice as attending physicians, factors may come into play that were not apparent to them when they were in training that ultimately affect their decisions to continue clinical EM practice. Although our study was not designed to shed further light on this issue, it is well established from previous research that female physicians experience workplace inequities compared with their male peers (eg, salaries,^28^ advancement,^29^ harassment, and discrimination^30^) and that these factors may play a role in their earlier attrition.

### Limitations

This study has several limitations. First, although the survey had a high response rate (97.4%), a substantial proportion of responses were missing for certain variables, which may affect the generalizability of subgroup analyses. Second, participation was voluntary and may be subject to response and social desirability bias, particularly in self-reported measures of burnout, educational debt, and career intentions. A third limitation is that the survey was cross-sectional and captured career plans at a single point in time during residency. It is unknown how closely these intentions align with actual career trajectories after graduation, although the ABEM In-Training Examination is administered during the end of February of each year, only 4 months before graduation. Fourth, while we identified associations between resident characteristics and career decision factors, the observational nature of this study precludes causal inferences.

## Conclusions

In this national survey study of graduating US EM residents, most planned to enter community-based or hybrid practice, with a minority of residents planning to practice in a rural setting. Lifestyle, geography, professional fulfillment, and salary expectations were the most important factors in career decision-making, while concerns about a potential workforce surplus and the COVID-19 pandemic were the least important. Burnout and educational debt were common, and both were associated with how residents prioritized career-related factors. Most residents anticipated practicing clinical EM for approximately 2 decades. These findings provide timely insights into the perspectives of the next generation of EPs and highlight important considerations for workforce planning and career sustainability initiatives in EM.

## Notes

Supplement 1. **eFigure 1.** Resident Characteristics Associated With Prioritizing Lifestyle in Career Decisions **eFigure 2.** Resident Characteristics Associated With Prioritizing Rural Practice Setting in Career Decisions **eFigure 3.** Resident Characteristics Associated With Prioritizing Professional Fulfillment in Career Decisions **eFigure 4.** Resident Characteristics Associated With Prioritizing Salary in Career Decisions **eFigure 5.** Resident Characteristics Associated With Prioritizing Burnout in Career Decisions **eFigure 6.** Resident Characteristics Associated With Prioritizing Family Expectations in Career Decisions **eFigure 7.** Resident Characteristics Associated With Prioritizing Influence of Mentor/Role Model in Career Decisions **eAppendix.** 2023 ITE Survey Questions

Supplement 2.
Data Sharing Statement
