# Supplementary material for: Career Plans Among Graduating US Emergency Medicine Residents
Source: JAMA Netw Open. 2026 Jan 27;9(1):e2555376. doi: 10.1001/jamanetworkopen.2025.55376 (PMC12848626; doi:10.1001/jamanetworkopen.2025.55376)
Supplement: Supplement 1. — eFigure 1. Resident Characteristics Associated With Prioritizing Lifestyle in Career Decisions eFigure 2. Resident Characteristics Associated With Prioritizing Rural Practice Setting in Career Decisions eFigure 3. Resident Characteristics Associated With Prioritizing Professional Fulfillment in Career Decisions eFigure 4. Resident Characteristics Associated With Prioritizing Salary in Career Decisions eFigure 5. Resident Characteristics Associated With Prioritizing Burnout in Career Decisions eFigure 6. Resident Characteristics Associated With Prioritizing Family Expectations in Career Decisions eFigure 7. Resident Characteristics Associated With Prioritizing Influence of Mentor/Role Model in Career Decisions eAppendix. 2023 ITE Survey Questions [file jamanetwopen-e2555376-s001.pdf]

Supplementary Online Content

Lu DW, Gu B, Courtney DM, et al. Career plans among graduating US emergency medicine residents. *JAMA Netw Open.* 2026;9(1):e2555376. doi:10.1001/jamanetworkopen.2025.55376

eFigure 1. Resident Characteristics Associated With Prioritizing Lifestyle in Career Decisions

eFigure 2. Resident Characteristics Associated With Prioritizing Rural Practice Setting in Career Decisions

eFigure 3. Resident Characteristics Associated With Prioritizing Professional Fulfillment in Career Decisions

eFigure 4. Resident Characteristics Associated With Prioritizing Salary in Career Decisions

eFigure 5. Resident Characteristics Associated With Prioritizing Burnout in Career Decisions

eFigure 6. Resident Characteristics Associated With Prioritizing Family Expectations in Career Decisions

eFigure 7. Resident Characteristics Associated With Prioritizing Influence of Mentor/Role Model in Career Decisions

eAppendix. 2023 ITE Survey Questions

This supplementary material has been provided by the authors to give readers additional information about their work.

© 2026 Lu DW et al. *JAMA Network Open.*

eFigure 1. Resident Characteristics Associated With Prioritizing Lifestyle in Career Decisions

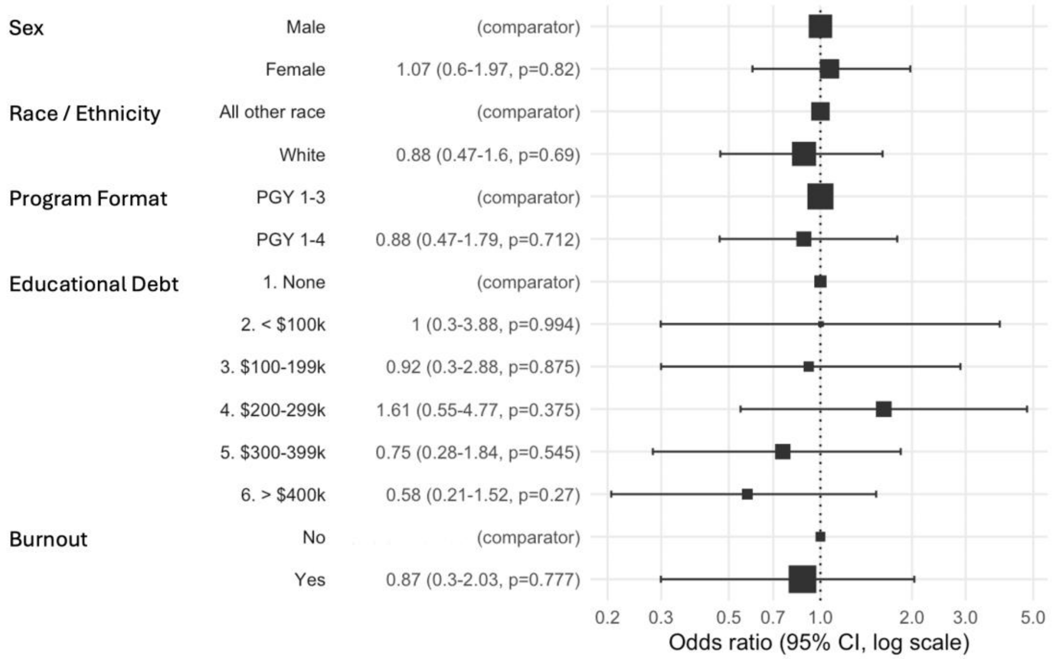

Abbreviations: CI (confidence interval), PGY (post-graduate year)

eFigure 2. Resident Characteristics Associated With Prioritizing Rural Practice Setting in Career Decisions

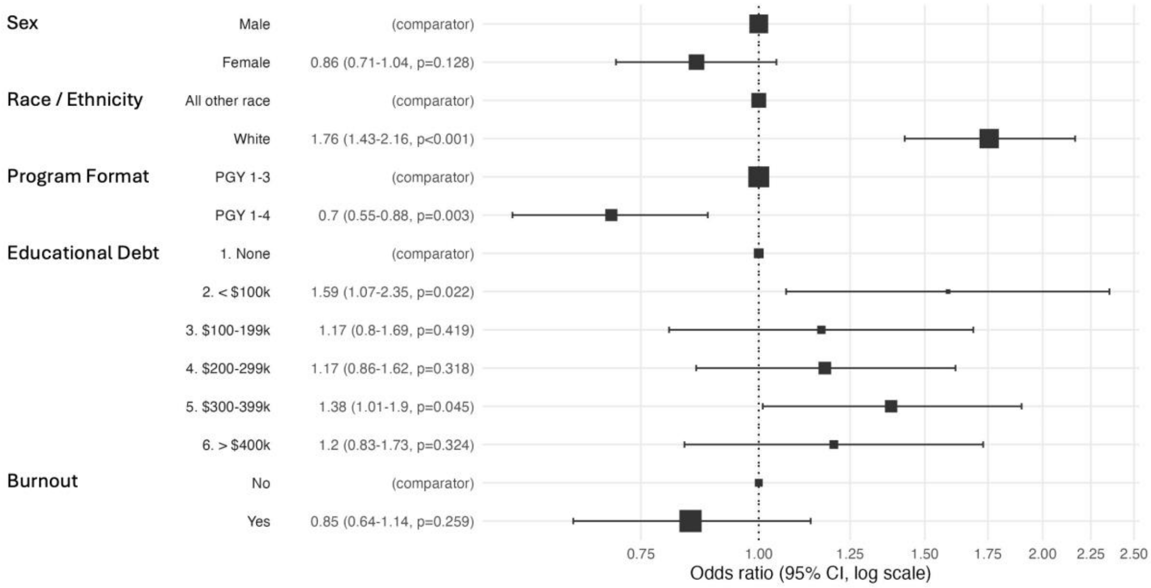

Abbreviations: CI (confidence interval), PGY (post-graduate year)

© 2026 Lu DW et al. *JAMA Network Open.*

eFigure 3. Resident Characteristics Associated With Prioritizing Professional Fulfillment in Career Decisions

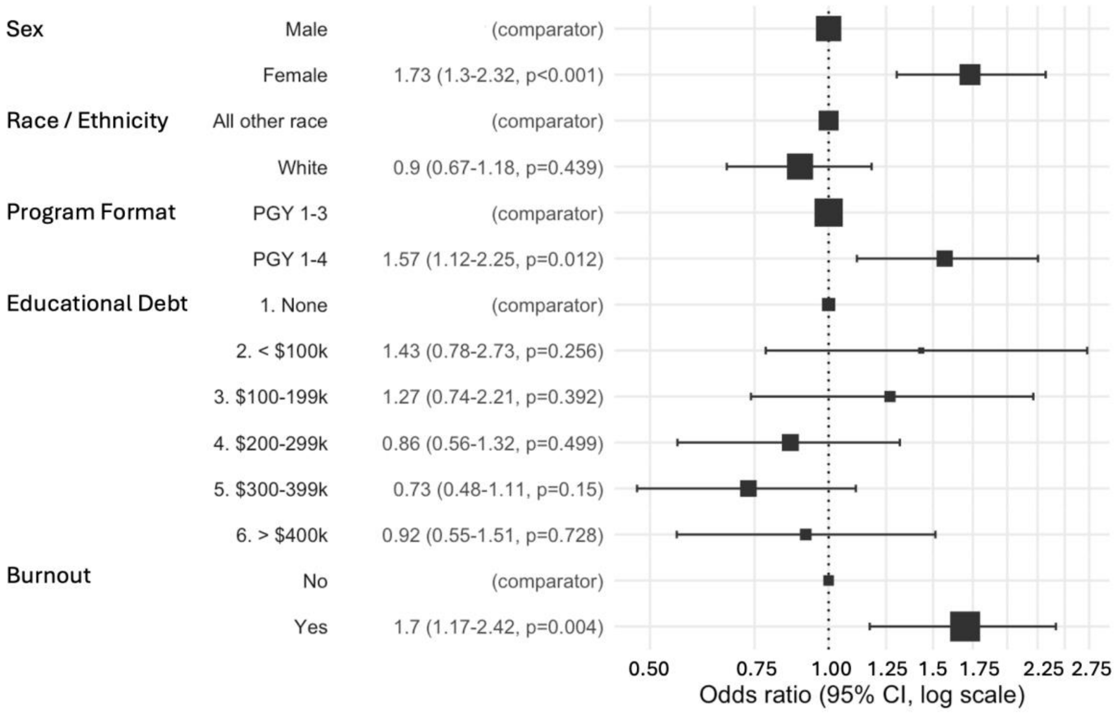

Abbreviations: CI (confidence interval), PGY (post-graduate year)

© 2026 Lu DW et al. *JAMA Network Open.*

eFigure 4. Resident Characteristics Associated With Prioritizing Salary in Career Decisions

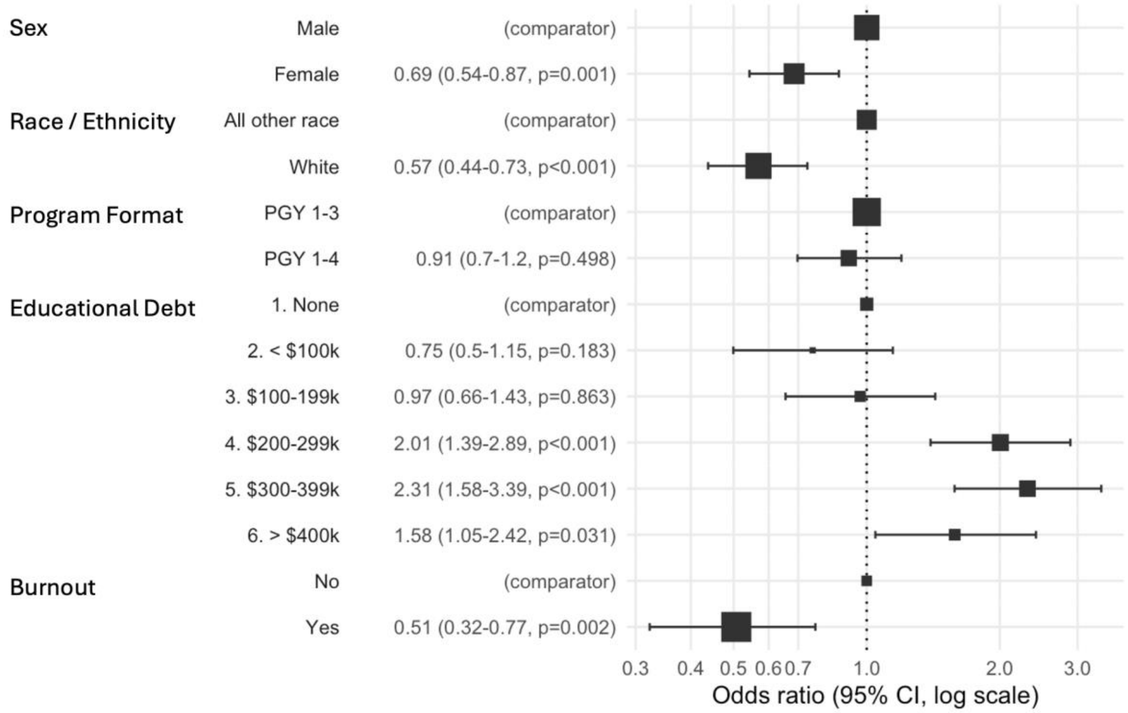

Abbreviations: CI (confidence interval), PGY (post-graduate year)

© 2026 Lu DW et al. *JAMA Network Open.*

eFigure 5. Resident Characteristics Associated With Prioritizing Burnout in Career Decisions

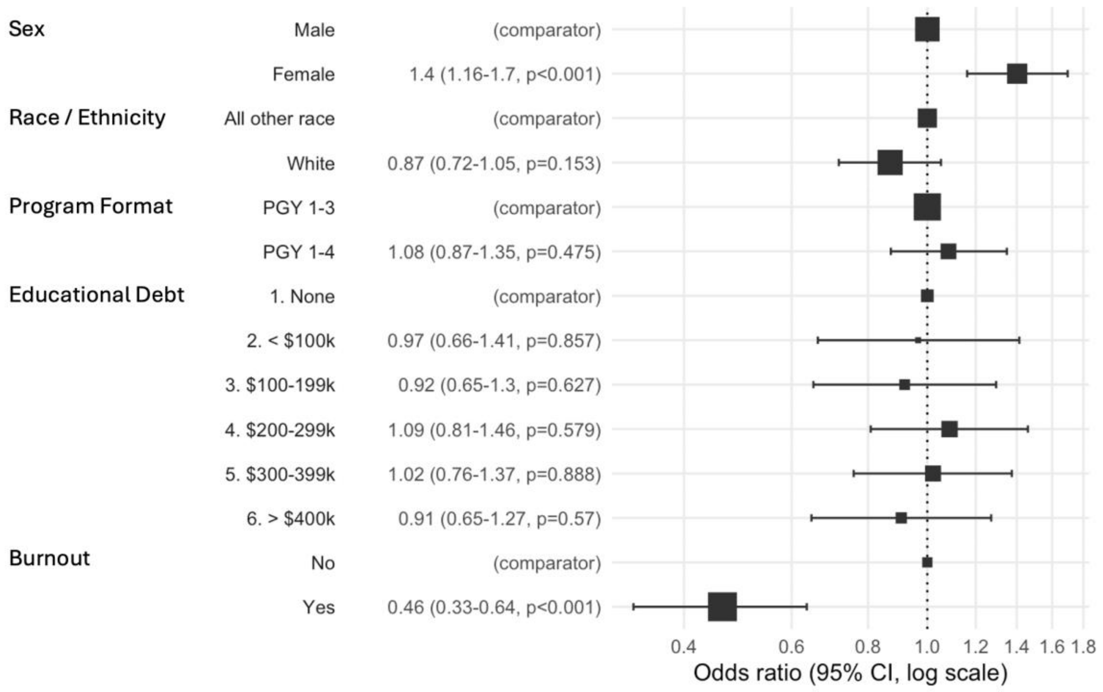

Abbreviations: CI (confidence interval), PGY (post-graduate year)

© 2026 Lu DW et al. *JAMA Network Open.*

eFigure 6. Resident Characteristics Associated With Prioritizing Family Expectations in Career Decisions

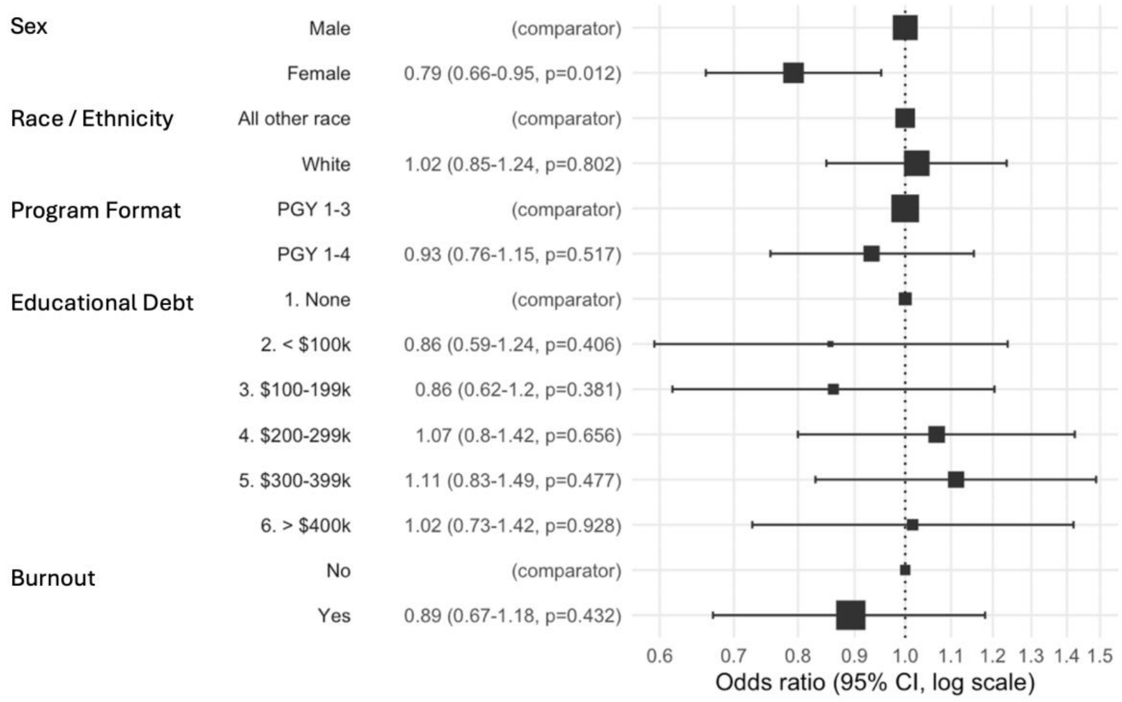

Abbreviations: CI (confidence interval), PGY (post-graduate year)

© 2026 Lu DW et al. *JAMA Network Open.*

eFigure 7. Resident Characteristics Associated With Prioritizing Influence of Mentor/Role Model in Career Decisions

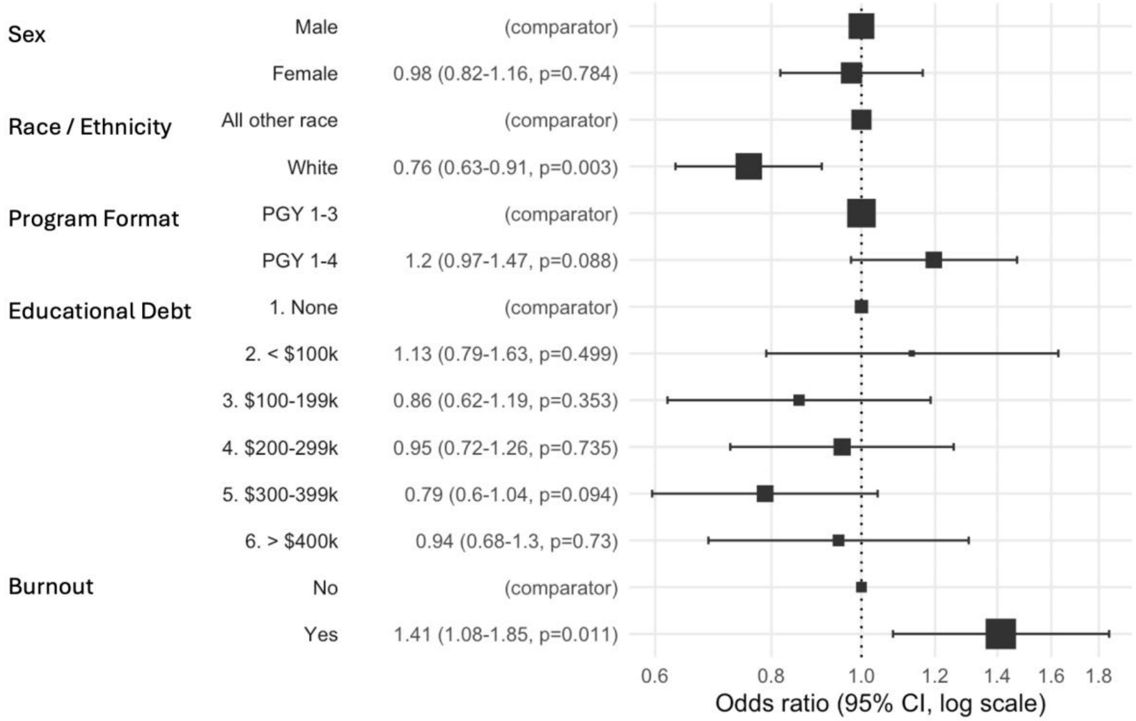

Abbreviations: CI (confidence interval), PGY (post-graduate year)

© 2026 Lu DW et al. *JAMA Network Open.*

eAppendix. 2023 ITE Survey Questions

DEMOGRAPHICS

1. I identify myself as: MC - select one
    a. Female
    b. Male
    c. Gender non-binary

2. The following best describes my ethnicity: MC - select one
    a. American Indian or Alaska Native
    b. Asian - Chinese
    c. Asian - Filipino
    d. Asian - Indonesian
    e. Asian - Japanese
    f. Asian - Indian
    g. Asian - Korean
    h. Asian - Bangladeshi
    i. Asian - Cambodian
    j. Asian - Laotian
    k. Asian - Pakistani
    l. Asian - Vietnamese
    m. Asian - Other Asian
    n. Black or African American - African American
    o. Black or African American - Afro-Caribbean
    p. Black or African American - African
    q. Black or African American - Other Black
    r. Hispanic, Latino, or of Spanish Origin - Argentinean
    s. Hispanic, Latino, or of Spanish Origin - Colombian
    t. Hispanic, Latino, or of Spanish Origin - Cuban
    u. Hispanic, Latino, or of Spanish Origin - Dominican
    v. Hispanic, Latino, or of Spanish Origin - Mexican
    w. Hispanic, Latino, or of Spanish Origin - Peruvian
    x. Hispanic, Latino, or of Spanish Origin - Puerto Rican
    y. Hispanic, Latino, or of Spanish Origin - Other Hispanic
    z. Middle Eastern/North African
    aa. Multi-Racial
    bb. Native Hawaiian or Other Pacific Islander - Guamanian
    cc. Native Hawaiian or Other Pacific Islander - Native Hawaiian
    dd. Native Hawaiian or Other Pacific Islander - Samoan
    ee. Native Hawaiian or Other Pacific Islander - Other Pacific Islander
    ff. Other
    gg. Unknown Race/Ethnicity
    hh. White

© 2026 Lu DW et al. *JAMA Network Open.*

EDUCATIONAL DEBT

3. What is the range of your educational debt? MC – select one
    a. None
    b. <$50K
    c. $50-99K
    d. $100-$149K
    e. $150-$199K
    f. $200-$249K
    g. $250-$299K
    h. $300-349K
    i. $350 – 399K
    j. >$400K

BURNOUT

Definition: <u>Personal burnout</u> is a state of prolonged physical and psychological exhaustion.

4. Thinking back on this academic year (July 2022 to present) and **<u>personal</u> burnout**: Likert Grid

	Always	Often	Sometimes	Seldom	Never/ Almost Never
a. How often do you feel tired?					
b. How often are you physically exhausted?					

Definition: <u>Work burnout</u> is a state of prolonged physical and psychological exhaustion, which is perceived as related to the person's work.

5. Thinking back on this academic year (July 2022 to present) and **<u>work</u> burnout**: Likert

	To a very high degree	To a high degree	Somewhat	To a low degree	To a very low degree
a. Do you feel burned out because of your work?					

6. Thinking back on this academic year (July 2022 to present) and **<u>work</u> burnout:** Likert

© 2026 Lu DW et al. *JAMA Network Open.*

	Always	Often	Sometimes	Seldom	Never/almost never
a. Do you feel worn out at the end of the working day?					

Definition: Patient burnout is a state of prolonged physical and psychological exhaustion, which is perceived as related to the person's work with patients.

7. Thinking back on this academic year (July 2022 to present) and **patient burnout**: Likert grid

	To a very high degree	To a high degree	Somewhat	To a low degree	To a very low degree
a. Do you find it hard to work with patients?					
b. Do you find it frustrating to work with patients?					

CAREER PLANS

8. What are your career plans after completing residency training? MC – select one

- Fellowship or additional education/training
- Practice primarily in an academic medical center/teaching hospital
- Practice primarily in a community-based hospital affiliated with an academic medical center
- Practice primarily in a community-based hospital
- Practice primarily in a Veterans Affairs hospital
- Practice primarily in an urgent care setting
- Active military practice
- Locum Tenens
- Other
- Unsure

9. In what type of setting do you intend to practice primarily after completing residency or fellowship training? MC – select one

- Urban (metropolitan area core with a population greater than 50,000)
- Large rural (large town core with a population between 10,000-50,000)

© 2026 Lu DW et al. *JAMA Network Open.*

- Small rural (towns with populations of 2,500-10,000 or areas without an urban core population of at least 2,500)

10. How important are the following factors in determining your career choice? *Likert grid: Strong influence, Moderate influence, Minor influence, No influence)*

    - Lifestyle
    - Competitiveness or prestige of career path
    - Mentor / role model influence
    - Educational debt or other financial responsibilities
    - Salary expectations
    - Family expectations
    - Geographic location
    - Burnout
    - Professional fulfillment
    - Prospect of a surplus of EM physicians by 2030
    - COVID-19 pandemic

PLANNED ATTRITION

11. How many years do you expect to practice clinical EM? Text box

12. When you cut back or leave clinical EM, what do you plan to do? (Please select all that apply.) Multiple select
    - Retire
    - Non-traditional EM clinical practice (e.g., urgent care, transitional primary care, telemedicine, observational medicine, etc.)
    - Administration
    - Education
    - Research
    - Obtain additional medical education or training
    - Work in a different career other than medicine
    - Other

© 2026 Lu DW et al. *JAMA Network Open.*
