# Supplementary material for: Career Plans Among Graduating US Emergency Medicine Residents
Source: JAMA Netw Open. 2026 Jan 27;9(1):e2555376. doi: 10.1001/jamanetworkopen.2025.55376 (PMC12848626; doi:10.1001/jamanetworkopen.2025.55376)
Supplement: Supplement 2. — Data Sharing Statement [file jamanetwopen-e2555376-s002.pdf]

Data Sharing Statement

Lu. Career Plans Among Graduating US Emergency Medicine Residents. *JAMA Netw Open*. Published January 27, 2026. doi:10.1001/jamanetworkopen.2025.55376

Data
Data available: No
